# Angiotensin II type 1 receptor antibodies as a contributor to microvascular inflammation in kidney transplant recipients: insights from statistical and artificial intelligence based approaches

**DOI:** 10.3389/fimmu.2025.1659939

**Published:** 2025-10-16

**Authors:** Jakub Mizera, Piotr Donizy, Agnieszka Halon, Dariusz Janczak, Marta Kepinska, Maciej Pondel, Miroslaw Banasik

**Affiliations:** ^1^ Department of Nephrology, Transplantation Medicine and Internal Diseases, Institute of Internal Diseases, Wroclaw Medical University, Wroclaw, Poland; ^2^ University Clinical Hospital in Wroclaw, Wroclaw, Poland; ^3^ Department of Clinical and Experimental Pathology, Wroclaw Medical University, Wroclaw, Poland; ^4^ Department of Vascular, General and Transplant Surgery, Wroclaw Medical University, Wroclaw, Poland; ^5^ Department of Pharmaceutical Biochemistry, Faculty of Pharmacy, Wroclaw Medical University, Wroclaw, Poland; ^6^ Department of Business Intelligence in Management, Wroclaw University of Economics and Business, Wroclaw, Poland

**Keywords:** kidney transplantation, artificial intelligence, angiotensin II type 1 receptor antibodies, Non-HLA, microvascular inflammation

## Abstract

**Introduction:**

In recent years, advancements in the classification of renal allograft pathology have led to a notable increase in the diagnosis of antibody-mediated rejection (ABMR). Particular attention has been given to microvascular inflammation (MVI), a subcategory of ABMR that was reappraised in the Banff 2022 classification. Recognizing a significant discrepancy between the number of patients testing positive for donor-specific anti-HLA antibodies (DSAs) and those clinically diagnosed with ABMR, this study aims to explore the potential role of non-HLA antibodies, specifically, antibodies against the angiotensin II type 1 receptor (AT1R), in the development of MVI.

**Material and methods:**

A retrospective analysis was performed on clinical and pathological data from 167 kidney transplant recipients. MVI was diagnosed histologically based on biopsy findings, specifically a glomerulitis score (g) > 0 and/or peritubular capillaritis (ptc) > 0. Based on these criteria, two patient cohorts were identified: 88 patients without MVI and 79 patients with MVI. Statistical analyses were conducted using appropriate methods, including chi-square tests, Student’s t-tests, and Mann–Whitney U tests. Additionally, complementary analyses utilizing artificial intelligence techniques such as correlation analysis, logistic regression and association rule mining were applied to maximize insights from the dataset.

**Results:**

Patients with MVI demonstrated a statistically significantly higher prevalence of AT1R antibodies compared to those without MVI. The finding was confirmed by both traditional statistical methods and artificial intelligence analysis. Furthermore, the MVI-positive cohort exhibited a higher frequency of C4d positivity compared to patients without MVI.

**Conclusions:**

The presence of AT1R antibodies may be associated with the development of microvascular inflammation, potentially contributing to allograft injury in a subset of cases of kidney transplant recipients. High AT1R abs titers (>12 U/ml) were particularly important, while lower levels did not show the meaningful association. These findings underscore the importance of broadening immunologic surveillance beyond conventional anti-HLA antibody screening.

## 1 Introduction

The Banff classification is widely regarded as the gold standard for diagnosing kidney transplant pathology. Since its introduction, it has undergone periodic revisions to incorporate new insights and developments in kidney pathology. The most recent update, published in 2022, introduced significant changes, particularly in the diagnostic criteria for antibody-mediated rejection (ABMR) (1). One of the key modifications in the 2022 Banff classification was the redefinition of microvascular inflammation (MVI), a subcategory of ABMR. As a result, numerous cases that were previously classified as non-rejection under the 2019 criteria are now categorized as probable ABMR or MVI (2). Consequently, ABMR, once considered a less prominent cause of kidney transplant rejection, has emerged as one of the most frequently diagnosed causes of immunological graft damage (2, 3).

This shift has prompted increased interest in understanding the factors contributing to MVI. A recent report published in *The New England Journal of Medicine* highlighted the clinical significance of this reclassification (2). Patients initially diagnosed as non-rejection under the 2019 Banff criteria, but later reclassified as MVI, DSA-negative, and C4d-negative under the 2022 criteria, were shown to have poorer graft survival compared to those whose diagnosis remained unchanged (1, 2, 4).

Traditionally, ABMR has been primarily associated with donor-specific antibodies (DSAs) directed against human leukocyte antigens (HLA) and with C4d-positivity. However, increasing evidence indicates a substantial discrepancy between ABMR-associated histologic findings and the detection of HLA-DSAs or C4d. This has led to growing interest in the role of non-HLA antibodies in ABMR pathogenesis (1, 5).

Among the non-HLA antibodies, those directed against the angiotensin II type 1 receptor (AT1R) have gained particular attention. Emerging data suggest that AT1R antibodies (AT1R abs) can directly induce endothelial activation and contribute to MVI development, even in the absence of detectable HLA-DSAs (6). As our understanding of ABMR and MVI increasingly focuses on endothelial injury and innate immune activation, AT1R abs are now being considered as potential drivers of these processes (7).

Our previous investigations have shown that AT1R abs are associated with more severe episodes of acute rejection and poorer graft function, even when HLA-DSAs are not present (8–10). At the time of that study, before the reappraisal of MVI in the Banff classification, the concept of MVI remained underestimated. Interestingly, many of the vascular lesions and rejection patterns observed in AT1R abs-positive patients now retrospectively align with what is defined as MVI under the 2022 update (1). This provides a strong rationale for re-evaluating the clinical relevance of AT1R abs in DSA-negative MVI cases, especially where traditional ABMR markers are absent (8).

In this study, we aimed to assess whether patients exhibiting histopathological features of MVI are more frequently positive for non-HLA antibodies, with a particular focus on angiotensin II type 1 receptor antibodies. To the best of our knowledge, this is the first study analyzing this phenomenon in the context of the reappraised MVI criteria introduced in the latest Banff classification. Understanding the role of AT1R abs in the development and progression of MVI is crucial for improving diagnostic accuracy, refining patient risk stratification, and guiding the development of targeted therapeutic strategies in kidney transplantation. Such advancements may ultimately enhance graft longevity and improve patient quality of life.

Given the complexity of the issue and recent advancements in data analysis, we extended our methodology beyond conventional statistical approaches by incorporating artificial intelligence techniques (11). As detailed in the Materials and Methods section, these advanced tools enabled a more comprehensive exploration and interpretation of the collected data.

## 2 Material and methods

### 2.1 Sample handling and ethics statement

The study was reviewed by the Bioethical Committee of Wroclaw Medical University, and due to its retrospective nature the need for approval was waived. At the same time, the Committee confirmed that the study design complies with the Declaration of Helsinki. Certificate number 102/2025.

The biopsy data were obtained from the Department of Pathology at the University Clinical Hospital of Wroclaw, where analyses of transplanted kidneys were conducted. Biopsies were performed in the past in response to deteriorating graft function, indicated by an increase in serum creatinine levels by at least 0,3 mg/dL and/or the occurrence of proteinuria. After collecting all specimens were paraffin-embedded and stored in the hospital archives. All specimens were reviewed by experienced renal pathologists (PD and AH) following standardized protocols (Paraffin embedded tissues were sliced into 5-micrometer-thick sections, then the samples were stained by: haematoxylin and eosin (H&E), periodic acid-Schiff (PAS), Jones methenamine silver (JMS), Massons’ trichrome, and immunohistochemically for C4d) to ensure consistent and reproducible histopathological interpretations. Tissue evaluations were based on the 2022 Banff Classification of Renal Allograft Pathology, with assessments performed in the glomerular, interstitial, tubular, vascular, and peritubular capillary compartments (1). Each compartment was scored on a standardized scale ranging from 0 (no lesions) to 3 (severe lesions), reflecting the severity of the observed pathological changes. All biopsies were performed between 2011 and 2019. Due to the retrospective nature of the study, the time from transplantation to biopsy was not standardized and varied across individuals.

### 2.2 Examined population – retrospective analysis, inclusion criteria

We conducted a retrospective analysis of archival medical records from the University Clinical Hospital in Wroclaw, Poland. The study included patients who met the following criteria:

- Underwent kidney transplantation.
- Had at least one biopsy of the transplanted kidney, subsequently evaluated by a pathologist according to the Banff classification.
- Underwent serum assessment for the presence of angiotensin II type 1 receptor antibodies.
- Had available medical history including routinely assessed immunological and non-immunological parameters.

**Figure 1:**
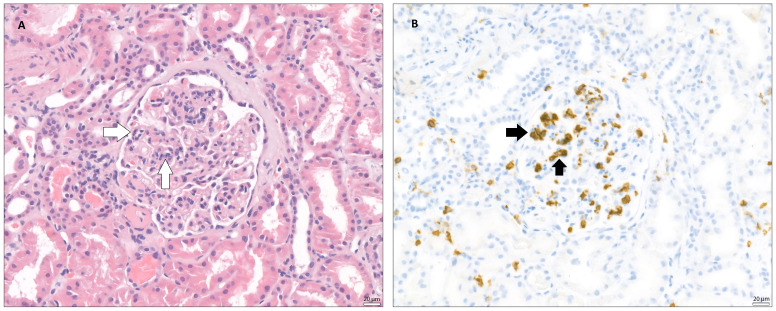
Glomerulitis (microvascular inflammation) – The images taken via light microscope both demonstrate a glomerulus from a transplanted kidney, stained with **(A)** - hematoxylin and eosin (H&E) and **(B)** – immunohistochemically (IHC) for CD45 (leukocyte common antigen, LCA). In both images, within the glomerular tuft, a segmental infiltration of inflammatory cells, mainly neutrophils and mononuclear leukocytes, can be observed, suggesting glomerulitis. White arrows indicate inflammatory infiltration within glomerular tuft. Black arrows indicate CD45 positivity, marking the position of leukocytes in the glomerulus.

**Figure 2:**
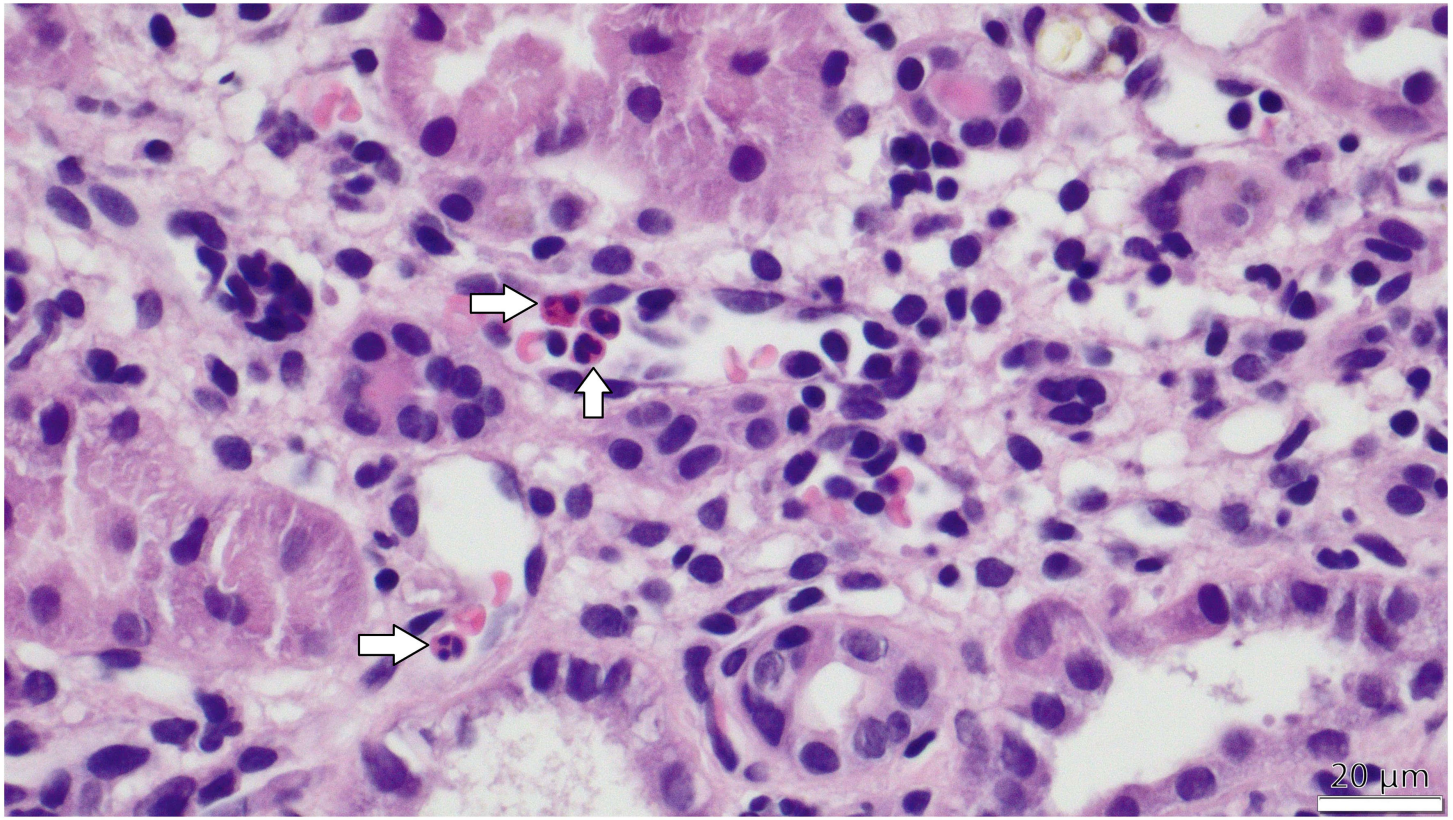
PTC-itis (microvascular inflammation) -The image taken via light microscope demonstrates a section of the renal cortex from a transplanted kidney, stained with hematoxylin and eosin (H&E). Multiple peritubular capillaries are visible adjacent to renal tubules, showing prominent infiltration of inflammatory cells, primarily mononuclear leukocytes and scattered neutrophils, identifiable by their dark purple-stained nuclei within the capillary lumens. White arrows in the figure indicate the inflammatory cells within capillary lumens.

As a result, we identified 167 patient records that met the eligibility criteria for further analysis. The transplant procedures were performed between 2010 and 2018. All data obtained from medical records were fully anonymized in accordance with best practices for retrospective analyses, ensuring the protection of patient confidentiality and compliance with ethical standards. Based on biopsy data, the study population was stratified into two groups: one with microvascular inflammation (MVI) and the other without MVI. Microvascular inflammation was diagnosed according to the Banff Classification of Renal Allograft Pathology and was defined by the presence of glomerulitis (*g*) > 0 (**Figure 1**) and/or peritubular capillaritis (*ptc*) > 0 (**Figure 2**) (1). To ensure diagnostic specificity, cases were included only if these changes occurred in the absence of recurrent or *de novo* glomerulonephritis. Additionally, in the presence of acute T cell-mediated rejection (TCMR), borderline infiltrates, or infection, a *ptc* > 0 alone was not considered sufficient for MVI diagnosis; in such cases, *g* > 0 was required as well.

### 2.3 Assessment of angiotensin II Type 1 receptor antibodies, endothelial cell antibodies and endothelin-1 type A receptor antibodies

For antibody assessment, blood samples were obtained during the routine examinations, which means that no additional blood collection for antibodies assessment was necessary. Serum samples for antibody detection were examined prior to the biopsy (with blood collection no more than one day before the procedure) and one week post-procedure. The highest value obtained from these two time points was used for further analysis. This approach was a part of a screening protocol.

Venous blood was collected into sterile 2.7 mL serum separator tubes. All samples were obtained between 8:00 and 9:00 a.m. and patients were not required to fast prior to collection. Following collection, blood samples were centrifuged at 1500 × g for 10 minutes at room temperature. The resulting serum was subsequently frozen at –80°C until further analysis.

The concentration of AT1R antibodies in serum was determined using a commercially available enzyme-linked immunosorbent assay (ELISA) kit (CellTrend, Luckenwalde, Germany, catalog no. 12000), according to the manufacturer’s protocol (12). Briefly, the analysis was performed on precoated microtiter plates. Diluted serum samples (1:100) and standards were added to the wells and incubated for 2 hours at 2–8°C. This was followed by washing steps and detection of AT1R antibodies using a horseradish peroxidase (POD)-labeled anti-human IgG antibody (1:100). Colorimetric detection was performed using a 3,3′,5,5′-tetramethylbenzidine (TMB) substrate, with absorbance measured at 450 nm and a reference wavelength of 630 nm. Antibody concentrations were calculated using standard curves, and values exceeding 10 U/mL were considered positive. Each sample was assayed in duplicate, and the mean value of the two measurements was considered for subsequent analyses.

### 2.4 Statistical analysis

Statistical analysis was conducted using TIBCO Statistica^®^ version 13.3.0 (13). Group comparisons and associations between categorical and continuous variables were evaluated using appropriate statistical methods. The chi-square test was employed to assess relationships between categorical variables by determining whether observed frequencies significantly deviated from expected distributions (14). For continuous variables, the choice of statistical test was based on data distribution and variance homogeneity. The Student’s t-test was used to compare mean values between two independent groups when normality and homogeneity of variances were satisfied. If these assumptions were not met, the Mann-Whitney U test was applied as a non-parametric alternative to compare median values (15). These methodological considerations ensured the accurate and reliable interpretation of results. p-values, with a threshold of p<0.05 were considered statistically significant.

### 2.5 Artificial intelligence analysis

A combination of statistical and data mining techniques was employed, consisting of the following stages:

#### 2.5.1 Correlation analysis of AT1R abs presence and the occurrence of MVI and visual inspection using regression plot

To assess the strength and direction of the association between AT1R abs levels (continuous variable) and microvascular inflammation (binary variable), both Pearson and Spearman correlation coefficients were calculated. The Pearson method was used to evaluate linear relationships, while the Spearman method assessed monotonic associations. Statistical significance was evaluated using corresponding p-values, with a threshold of p<0.05 considered statistically significant.

The correlation analysis was performed in Python version 3.13 using the scipy.stats library (16).

To support the interpretation of the observed associations, a scatterplot with a fitted linear regression line was generated. This visualization helps illustrate how increasing levels of AT1R abs correspond with the distribution and density of MVI observations.

The scatterplot was generated in Python version 3.13 using the seaborn visualization library (17).

#### 2.5.2 Logistic regression modelling of relationship between AT1R abs levels and probability of MVI

A binary logistic regression model was constructed to quantify how changes in AT1R abs levels affect the probability of microvascular inflammation. The MVI variable was treated as a binary outcome (0=no inflammation, 1=presence of inflammation), and AT1R abs served as the independent predictor. Odds ratios (OR), confidence intervals, and p-values were reported to interpret the effect size and statistical significance of AT1R abs on the risk of inflammation.

The intercept represents the log-odds of inflammation when AT1R abs is zero. The β coefficient indicates the change in log-odds of inflammation per one-unit increase in AT1R abs. The p-value tests whether this effect differs significantly from zero. The odds ratio (OR), derived from exponentiating β, reflects the multiplicative change in odds of inflammation per unit increase in AT1R abs. Lastly, the 95% confidence interval (CI) for the OR provides a range of plausible values for this effect; if the interval excludes 1, the association is considered statistically significant.

These parameters help to better characterize the nature and direction of the association between AT1R abs levels and microvascular inflammation. Unlike simple correlation measures or univariate statistical tests, logistic regression captures the probability-based relationship between a continuous predictor and a binary outcome, offering insights into how risk evolves across the spectrum of AT1R abs values.

The logistic regression analysis was performed in Python version 3.13 using the statsmodels.api library (18).

#### 2.5.3 Receiver operating characteristic curve analysis – evaluation of diagnostic performance of anti-AT1R abs

A receiver operating characteristic (ROC) curve was constructed to evaluate the diagnostic performance of AT1R antibody levels for predicting microvascular inflammation. The ROC curve plots sensitivity (true positive rate) against 1 – specificity (false positive rate) across a range of possible cutoff values, providing a comprehensive view of how test performance changes with the threshold. The area under the curve (AUC) was calculated as a summary metric of discriminative ability; values close to 1.0 indicate excellent discrimination, whereas values near 0.5 suggest no better performance than chance. This approach allowed us to objectively assess the capacity of AT1R abs to distinguish between patients with and without microvascular inflammation.

The ROC curve analysis was performed in Python version 3.13 using the scikit-learn library (19).

#### 2.5.4 Association rule mining - quantile analysis of MVI likelihood depending on AT1R abs levels

To complement the inferential statistical approach, association rule mining was employed to uncover patterns in categorical groupings of AT1R abs values and their association with MVI:

- The continuous AT1R abs variable was discretized into five quantile-based bins (quintiles) to reflect relative antibody levels.
- One-hot encoding was applied to represent the membership of each observation in one of the five AT1R abs intervals, resulting in binary flags for each bin. The MVI variable was also binarized and included in the itemset.
- Using the Apriori algorithm, association rules were generated to assess the relationship between AT1R abs interval membership and the presence of MVI.
- Each rule was evaluated based on support, confidence, and lift:
  - Support measures how frequently the rule occurs in the dataset.
  - Confidence reflects the conditional probability of MVI given a specific AT1R abs range.
  - Lift indicates how much more likely MVI is to occur when a particular AT1R abs interval is present, compared to its baseline probability.

This method provided an interpretable framework to evaluate how the likelihood of microvascular inflammation increases across quantiles of AT1R abs.

Association rules were calculated in Python version 3.13 using the mlxtend library, and AT1R abs quantiles were computed using pandas.qcut() from the pandas library (20, 21).

## 3 Results

Following diagnostic criteria for MVI described in the preceding section, the study cohort comprised 88 patients without microvascular inflammation and 79 patients with microvascular inflammation. There were no statistically significant differences in sex distribution or mean recipient age between the two groups. Moreover, both the distribution of immunosuppressive protocols and the underlying causes of chronic kidney disease leading to transplantation were similar between the groups.

A detailed demographic characterization of the study population is presented below (**Table 1**).

**Table 1:** Detailed demographic characterization of the study population.

Feature	No MVI (n=88)	MVI (n=79)	*P* value
Recipient sex	female n=27	female n=32	NS
	male n=61	male n=47	
Recipient age at tx – years - median (range)	44.5 (11-71)	42 (16-74)	NS
Donor sex	female n=28	female n=28	NS
	male n=41	male n=37	
	un n=19	un n=14	
Donor age at tx -years - median (range)	53 (16-72)	46,5 (9-69)	NS
Primary recipient’s chronic kidney disease	DN n=3	DN n=4	NS
	GN n=36	GN n=31	
	LN n=3	LN n=3	
	HTN n=6	HTN n=10	
	PN n=3	PN n=1	
	PKD n=12	PKD n=6	
	IN n=4	IN n=6	
	OU n=4	OU n=2	
	un n=17	un n=16	
Immunosuppressive regimen following the transplantation	1 n=4	1 n=1	NS
	2 n=36	2 n=31	
	3 n=3	3 n=2	
	4 n=1	4 n=0	
	5 n=2	5 n=1	
	6 n=1	6 n=1	
	7 n=20	7 n=12	
	8 n=2	8 n=0	
	9 n=0	9 n=3	
	10 n=0	10 n=1	
	un n=19	un n=27	

MVI, microvascular inflammation; NS, not statistically significant; tx, transplantation; DN, diabetic nephropathy; GN, glomerulonephritis; LN, lupus nephropathy; HTN, hypertensive nephropathy; PN, pyelonephritis; PKD, polycystic kidney disease; IN, interstitial nephritis; OU, obstructive uropathy; un, other; unknown or not specified. Immunosuppressive regimens: 1- tacrolimus; mycophenolate mofetil; glucocorticosteroids; basiliximab; 2, tacrolimus; mycophenolate mofetil; glucocorticosteroids; 3- tacrolimus; mycophenolate mofetil; 4, tacrolimus; azathioprine; glucocorticosteroids; 5, tacrolimus; glucocorticosteroids; 6, cyclosporine A; mycophenolate mofetil; glucocorticosteroids; basiliximab; 7 - cyclosporine A; mycophenolate mofetil; glucocorticosteroids; 8, cyclosporine A; azathioprine; glucocorticosteroids; 9, cyclosporine A; everolimus; glucocorticosteroids; 10 - cyclosporine A; glucocorticosteroids.

Moreover, there were no statistically significant differences between analysed groups regarding the incidence of HLA-specific antibodies, in both I and II class. Complete analysis of HLA mismatches between donors and recipients was included, providing complementary information regarding the immunological risk profile of our cohort (**Table 2**).

**Table 2:** Results – differences between analysed populations.

Feature	No MVI (n=88)	MVI (n=79)	*P* value
Anti-AT1R abs	positive n=21	positive n=37	**0.0018**
	negative n=67	negative n=42	
CIT (h); median (range)	23 (0-44)	21 (2-37)	NS
WIT (min); median (range)	15 (0-50)	27 (0-60)	0.01
Max PRA; mean (range)	8 (0-99)	14.52 (0-86)	NS
HLA A mismatch; mean (range)	1.23 (0-2)	1.32 (0-2)	NS
HLA B mismatch; mean (range)	1.4 (0-2)	1.47 (0-2)	NS
HLA DR mismatch; mean (range)	0.89 (0-2)	1.07 (0-2)	NS
Total mismatch; mean (range)	3.51 (0-6)	3.86 (0-6)	NS
Anti-HLA abs class I (% of population with positive result)	26	25	NS
Anti-HLA abs class II (% of population with positive result)	28	34	NS
Anti-AECA abs	positive n=18	positive n=15	NT
	negative n=50	negative n=25	
	un n=20	un n=39	
Anti-ETAR abs	positive n=14	positive n=18	NS
	negative n=71	negative n=58	
	un n=3	un n=3	
Anti-AT1R/AECA/ETAR	positive n=37	positive n=48	0.024
	negative n=51	negative n=31	
*i* score -biopsy; mean (range)	0.9 (0-3)	1.33 (0-3)	0.003
*t* score -biopsy; mean (range)	0.93 (0-3)	1.43 (0-3)	0.0009
*v* score -biopsy; mean (range)	0.14 (0-2)	0.48 (0-2)	**0.007**
C4d+	0 n=66	0 n=30	**0.000029**
	1 n=14	1 n=32	
	2 n=2	2 n=6	
	3 n=0	3 n=4	
	un n=6	un n=7	

MVI, microvascular inflammation; anti- AT1R abs - angiotensin II Type 1 receptor antibodies; CIT, cold ischemia time; WIT, warm ischemia time; NS, not statistically significant; PRA, panel of reactive antibodies; HLA, human leukocyte antigen; anti- AECA abs - anti-endothelial cell (AECA) antibodies; anti-ETAR abs- anti-endothelin type A receptor antibodies; NT, not tested for statistical significance. Particularly interesting findings were marked with bold numbers.

### 3.1 Statistical analysis

#### 3.1.1 Association between non-HLA antibodies and MVI

Among all analysed patients, angiotensin II Type 1 receptor (AT1R) antibodies were detected in 58 individuals, whereas 109 patients tested negative for their presence. The prevalence of AT1R antibodies was significantly higher in the group with microvascular inflammation, where 37 out of 79 patients (46.8%) tested positive, compared to the group without microvascular inflammation, in which 21 out of 88 patients (23.9%) were positive (p=0.0018) (**Table 2**).

Additionally, the study population was screened for other non-HLA antibodies, including anti-endothelial cell (AECA) antibodies and anti-endothelin type A receptor (ETAR) antibodies. An independent analysis was not conducted for anti-AECA due to a high number of missing values (59 unknown records across the entire cohort). In contrast, anti-ETAR data were largely complete, with only six missing records (3 for both groups). The threshold for a positive anti-ETAR result was set at >10 U/mL, consistent with the previously established cutoff for anti-AT1R positivity. In the subgroup with microvascular inflammation, 18 out of 76 patients (23.7%) were tested positive for anti-ETAR antibodies, compared to 14 out of 85 patients (16.5%) in the group without microvascular inflammation (**Table 2**). This difference did not reach statistical significance. When analyzing the combined presence of the tested antibodies (anti-AECA, anti-ETAR, and anti-AT1R), seropositivity for at least one was more frequently observed in patients with microvascular inflammation (48 out of 76; 63.2%) compared to those without microvascular inflammation (37 out of 88; 42%), with a statistically significant difference (p=0.024) (**Table 2**). However, given the substantial number of missing anti-AECA results, this finding should be regarded as intriguing but requiring further investigation to confirm its validity.

#### 3.1.2 Influence of cold and warm ischemia times on MVI incidence

The data pertaining to cold and warm ischemia times were analysed. While there were no statistically important differences with regard to cold ischemia time between analysed groups, we found out that kidneys which were exposed to longer warm ischemia time were statistically more often affected by microvascular inflammation (p=0,01) (**Table 2**).

#### 3.1.3 Histopathological findings across MVI cohort

A highly significant association was observed between microvascular inflammation and C4d positivity (p=0.000029). C4d deposition was assessed and categorized into four grades: 0 (no detectable C4d staining in peritubular capillaries), 1 (weak or focal staining, typically affecting <10% of peritubular capillaries), 2 (moderate staining, involving 10–50% of peritubular capillaries), and 3 (extensive C4d deposition, affecting >50% of peritubular capillaries). Notably, C4d positivity (grades 1, 2, and 3) was more frequently observed in patients exhibiting microvascular inflammation (**Table 2**).

Furthermore, patients with microvascular inflammation exhibited a higher frequency of histopathological alterations across all analyzed compartments (*i, t, v*) of the transplanted kidney. The mean histopathological scores, which quantify the severity of changes in the interstitium, tubules, and arterial vessels, were significantly elevated in individuals with microvascular inflammation, with p-values of 0.003, 0.0009, and 0.007, respectively (**Table 2**).

### 3.2 Artificial intelligence analysis

#### 3.2.1 Correlation analysis of AT1R abs presence and the occurrence of MVI and visual inspection using regression plot

**Figure 3:**
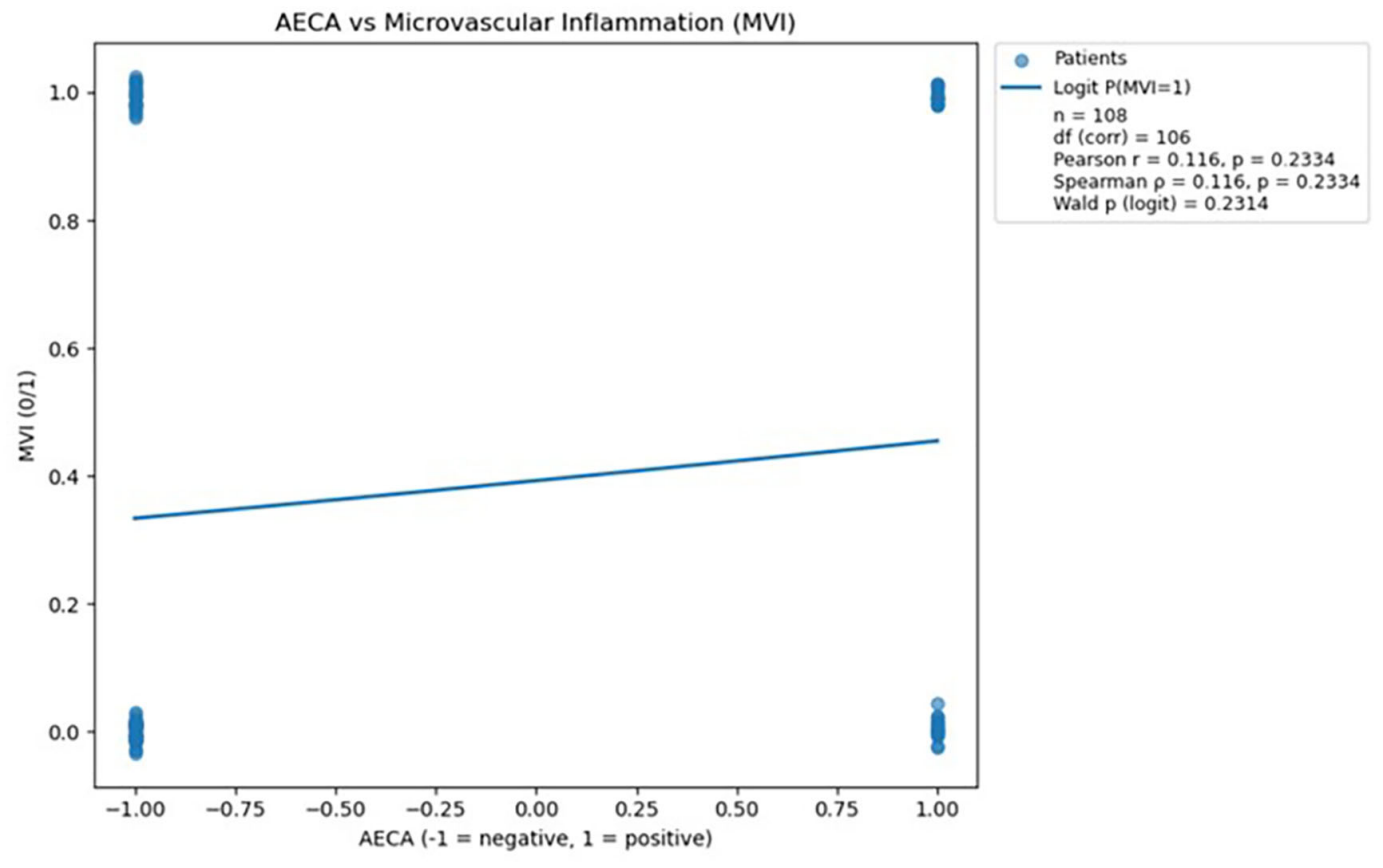
Scatterplot demonstrating the relationship between AECA antibody status and microvascular inflammation, with a fitted logistic regression line; no statistically significant association was observed (p > 0.05). The logistic regression analysis was performed in Python version 3.13 using the statsmodels.api library (18).

**Figure 4:**
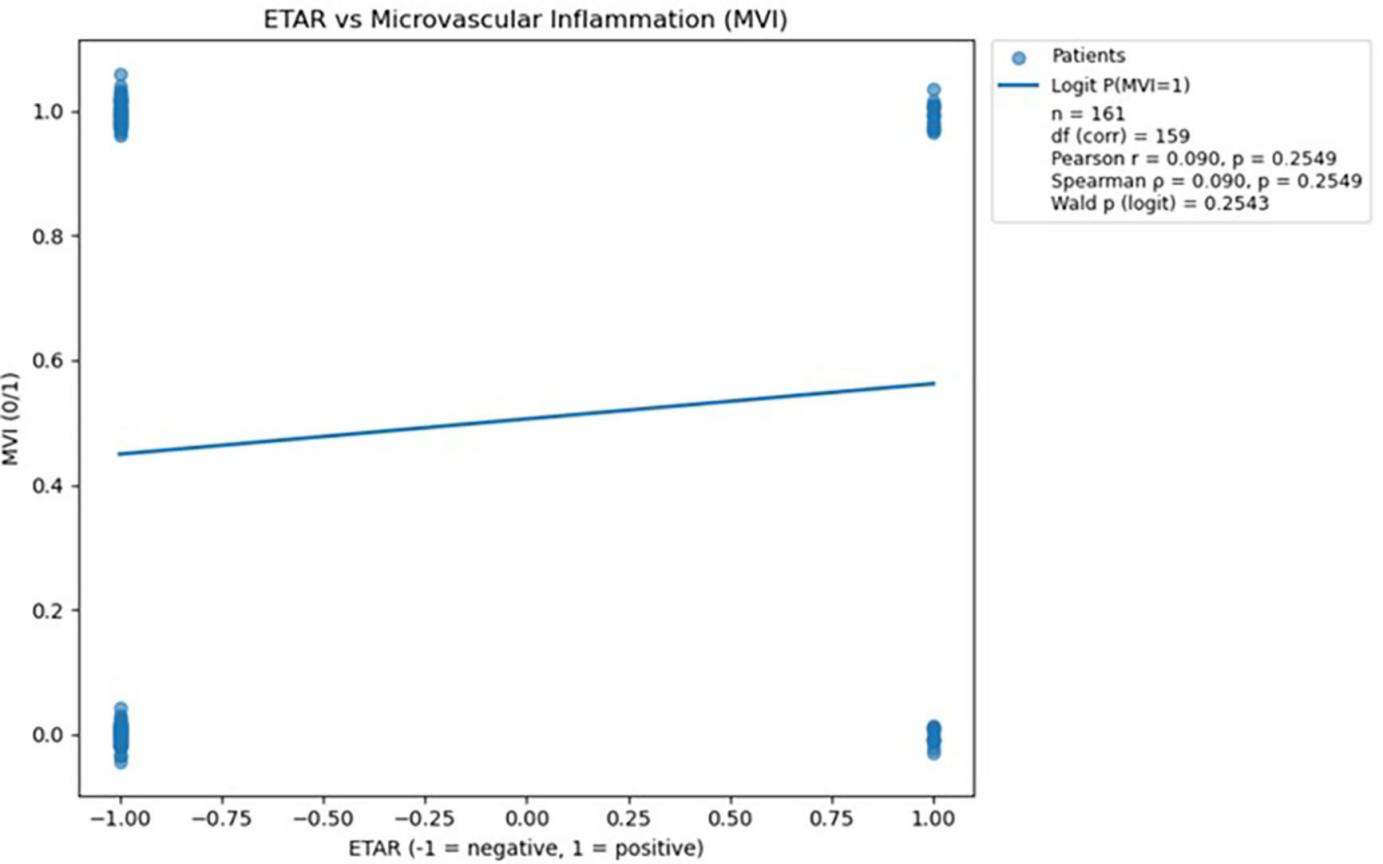
Scatterplot demonstrating the relationship between ETAR antibody status and microvascular inflammation, with a fitted logistic regression line; no statistically significant association was observed (p > 0.05). The logistic regression analysis was performed in Python version 3.13 using the statsmodels.api library (18).

**Figure 5:**
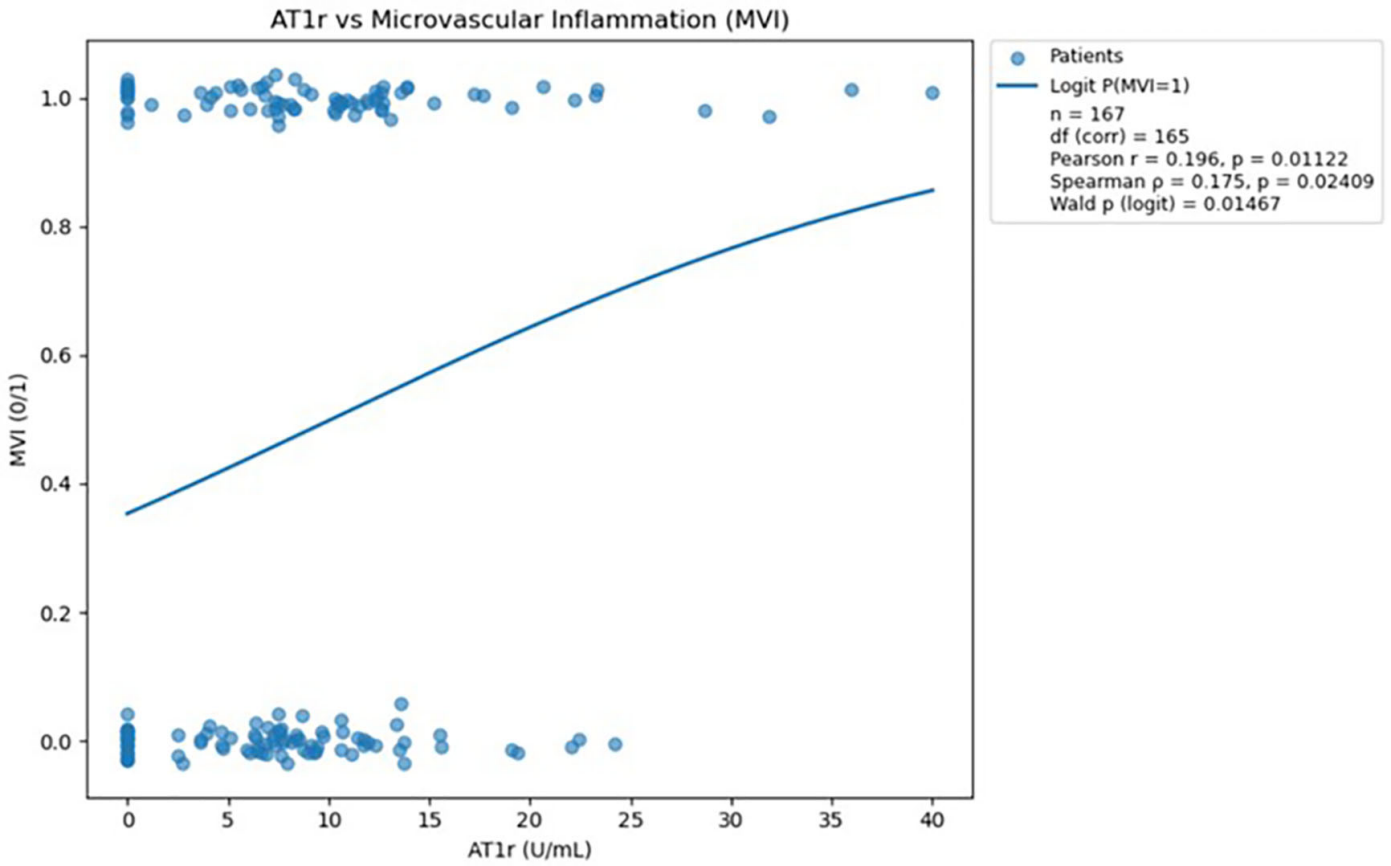
Scatterplot demonstrating the relationship between AT1R antibody levels and microvascular inflammation, with a fitted logistic regression curve; a statistically significant positive association was observed (p<0.05). The logistic regression analysis was performed in Python version 3.13 using the statsmodels.api library (18).

Three antibodies (anti-AT1R, anti-ETAR, and anti-AECA) were subjected to logistic regression and correlation analyses, with the resulting regression plots presented in **Figures 3**–**5**. For each antibody, Pearson’s and Spearman’s correlation coefficients with corresponding degrees of freedom were calculated to assess the strength and direction of linear and monotonic associations, while the Wald p-value from logistic regression was reported to evaluate the statistical significance of the relationship between antibody levels and the probability of microvascular inflammation.

Analysis of AECA antibodies showed no statistically significant association with microvascular inflammation, as indicated by both correlation tests and logistic regression (all *p* > 0.05). Consequently, AECA was not considered further in predictive modeling due to the lack of meaningful relationship with MVI.

Analysis of ETAR antibodies did not reveal a statistically significant association with microvascular inflammation, as both correlation coefficients and logistic regression results yielded *p*-values greater than 0.05. Therefore, ETAR was excluded from further predictive analysis due to its lack of meaningful relationship with MVI.

Analysis of AT1R antibodies demonstrated a statistically significant positive association with microvascular inflammation, confirmed by both correlation tests (Pearson and Spearman) and logistic regression (all *p*<0.05). These findings indicate that higher AT1R antibody levels are meaningfully linked to an increased probability of MVI.

Both Pearson and Spearman correlations (presented above in the figure) indicate a weak but statistically significant positive association between AT1R antibody levels and the occurrence of microvascular inflammation.

#### 3.2.2 Logistic regression modelling of relationship between AT1R abs levels and probability of MVI

The logistic regression model converged successfully after 5 iterations (final log-likelihood=0.6719), indicating stable model estimation.

The intercept was estimated at –0.6037, representing the baseline log-odds of microvascular inflammation when AT1R abs is zero. The coefficient for AT1R abs was 0.0596, suggesting that each unit increase in AT1R abs level is associated with a 0.0596 increase in the log-odds of inflammation.

This effect was statistically significant (p=0.0147), supporting a positive relationship between AT1R abs levels and the probability of microvascular inflammation. The corresponding odds ratio (OR) was 1.0614, indicating that with each unit increase in AT1R abs, the odds of inflammation increase by approximately 6.1%.

The 95% confidence interval for the odds ratio was [1.0118, 1.1135], which does not include 1.0, further confirming the significance of the association.

The visualization (**Figure 5**) presents a logistic regression model showing the predicted probability of microvascular inflammation (y-axis) across increasing AT1R abs levels (x-axis), with the red curve representing the fitted regression line and blue dots indicating observed binary outcomes.

#### 3.2.3 ROC curve analysis - evaluation of diagnostic performance of anti-AT1R abs

**Figure 6:**
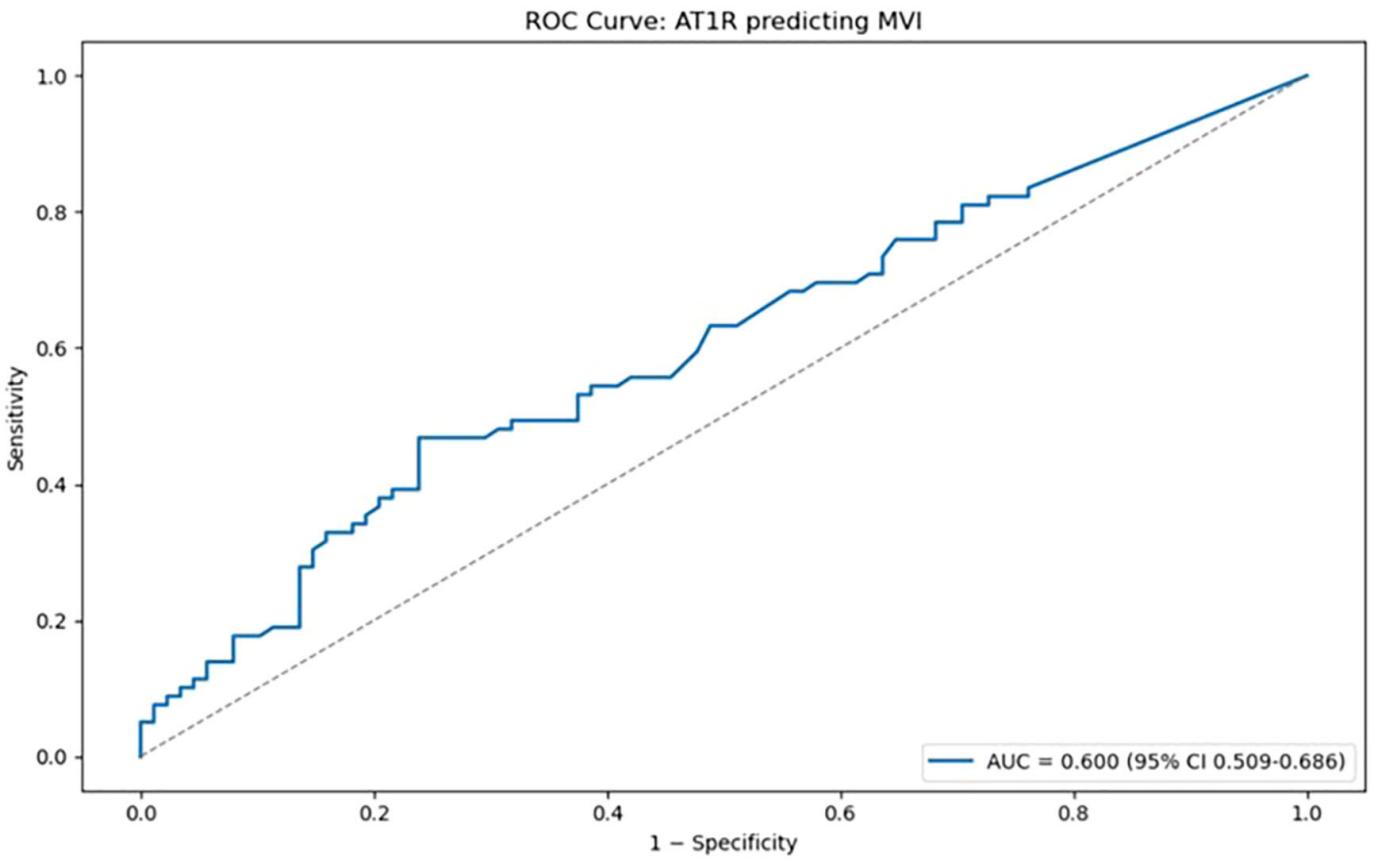
Receiver operating characteristic (ROC) curve illustrating the discriminatory performance of AT1R antibody levels for predicting microvascular inflammation (MVI). The area under the curve (AUC) was 0.600 (95% CI: 0.509–0.686), indicating limited predictive value. The ROC curve analysis was performed in Python version 3.13 using the scikit-learn library (19).

Receiver operating characteristic (ROC) curve was generated (**Figure 6**) to assess the discriminatory ability of AT1R antibody levels for predicting microvascular inflammation. The area under the curve (AUC) was 0.600 (95% CI: 0.509–0.686), indicating informative, yet modest predictive power. This analysis allowed us to further evaluate the potential of AT1R as a biomarker.

#### 3.2.4 Association rule mining (ARM)- quantile analysis of MVI likelihood depending on AT1R abs levels

To assess the relationship between AT1R antibody levels and the occurrence of microvascular inflammation (MVI), an association rule analysis was performed. The continuous variable AT1r was divided into five equally sized quantiles, each encoded as a binary variable (AT1R_q1 to AT1R_q5). Association rules were calculated with respect to the presence of microvascular inflammation (MVI=1), using the standard metrics: support, confidence, and lift. Association rules are presented in **Table 3**.

**Table 3:** Illustration of association rules.

AT1R_quantile	AT1R_range	Support	Confidence	Lift
AT1R_q5	(12.388, 40.0]	0.131737	0.647059	1.367833
AT1R_q4	(8.744, 12.388]	0.101796	0.515152	1.088991
AT1R_q2	(0.24, 6.8]	0.083832	0.411765	0.870439
AT1R_q3	(6.8, 8.744]	0.077844	0.406250	0.858782
AT1R_q1	[0.0, 0.24]	0.077844	0.382353	0.808265

Source: own elaboration in python/mlxtend library. Quantiles were ranked in descending order according to their lift values. Association rules were calculated in Python version 3.13 using the mlxtend library, and AT1R abs quantiles were computed using pandas.qcut() from the pandas library (20, 21).

ARM key findings:

- The highest AT1R abs quantile (AT1R_q5: 12.388–40.0) showed:
  o the highest support (13.2%),
  o the highest likelihood of MVI within the group (confidence=64.7%),
  o and the greatest lift (1.37), indicating that the probability of MVI was 37% higher in this group compared to the general population.
- Intermediate AT1R abs levels (AT1R_q2–q3: 0.24–8.744) were associated with:
  o lower confidence values (~41%),
  o and lift values below 1, suggesting that MVI was less likely in these quantiles than in the overall cohort.
- The lowest AT1R abs quantile (AT1R_q1: below 0.24) had the lowest confidence (38.2%) and a lift of 0.88, further indicating a decreased risk of MVI.

A clear positive trend between AT1R antibody levels and the probability of microvascular inflammation was observed. Notably, the highest AT1R abs quantile was strongly associated with an increased risk of inflammation, suggesting that AT1R abs may serve as a potential biomarker for MVI in kidney transplant recipients. However, the titers in lower quantiles did not show a meaningful association.

## 4 Discussion

The earliest investigations into the role of AT1R antibodies in kidney transplant recipients began in 00s on patient’s records from the early 1990s (22). Angiotensin II Type 1 Receptor antibodies have been found to exhibit variable associations with kidney transplant outcomes. Numerous studies have demonstrated that AT1R abs are associated with antibody-mediated rejection, even in the absence of HLA donor-specific antibodies. Furthermore, the presence of AT1R abs may synergize with HLA DSA, amplifying pathological changes, such as microvascular inflammation, which can be observed in biopsy (7, 23–25). Elevated pre-transplant levels of AT1R abs have been correlated with an increased risk of allograft loss after three years (26) Additionally, the *de novo* development of these antibodies following transplantation has been linked to graft failure (27).

In cases where AT1R abs were present, there were also findings of reduced graft function, lower estimated glomerular filtration rate over two years, and elevated inflammatory markers such as TNF-alpha, IL-1β, and IL-8 (28, 29). Furthermore, AT1R abs positivity in the context of indication biopsy was associated with ABMR and diminished allograft performance (30).

In our study, the prevalence of anti-AT1R abs was 23.86% in the non-MVI cohort and 46.84% in the MVI cohort, with an overall prevalence of 34.73% across the entire study population. Previous studies have reported a wide range of anti-AT1R abs positivity, varying from 21.4% in the general population to as high as 58% in pediatric cohorts. These findings indicate that the prevalence observed in our study is consistent with the range reported in the literature (28, 30).Despite these findings, some other studies have not confirmed a consistent association between AT1R abs and acute rejection episodes or graft failure (31). In some cases, AT1R abs status did not distinguish between patients with microvascular inflammation in the absence of HLA DSA and those with stable grafts (32). Additionally, pre-transplant AT1R abs levels did not always correlate with increased ABMR risk, and immunoassay results often failed to reflect actual antibody levels (32). These discrepancies suggest that while AT1R abs may contribute to adverse outcomes, their role is complex and may depend on context, particularly in combination with other immunological factors.

Our analysis of the incidence of anti-ETAR antibodies revealed no clear differences between patients with and without MVI. Nevertheless, previous studies have indicated that in cases of molecular ABMR, anti-ETAR antibodies may occur at a significantly higher frequency (33). Moreover, a high association between levels of anti-ETAR antibodies and diminished function of kidney graft was reported in the literature (29). The potential significance of anti-AECA antibodies was not assessed in our study due to the high proportion of missing data. However, they cannot be overlooked, as in other studies pre-transplant positivity was associated with increased rates of grade II TCMR and ABMR (34). Although our analysis did not demonstrate significant associations for these antibodies, the available evidence from the literature suggests that they should not be disregarded in scientific research.

In the light of reappraisal of microvascular inflammation by Banff 2022 criteria we believe that our findings are particularly important and provide new insights into understanding the role of non-HLA antibodies in kidney transplant recipients (1). Furthermore, a recent article by Sablik et al. supports the notion that incorporating additional rejection phenotypes is valuable for standardizing diagnostic approaches in kidney allograft evaluation (2). Hence, we suggest that exploring non-traditional possible rejection contributors not directly linked with anti-HLA antibodies is essential.

We demonstrated that AT1R abs can play an independent role in the development of microvascular inflammation in kidney allografts. In our study, a significantly higher prevalence of AT1R abs was observed in patients with microvascular inflammation despite similar HLA mismatch profiles, suggesting that these antibodies contribute to graft injury through mechanisms that are distinct from traditional HLA‐mediated alloimmunity. This observation is mostly in line with earlier reports that have highlighted the pathogenic role of AT1R abs in transplant rejection, with studies demonstrating that these antibodies not only can trigger endothelial activation and vascular inflammation but also may promote vasoconstriction and thrombosis, thereby accelerating graft injury (6, 35).

In addition to the immunologic factors, our results underscore the detrimental impact of prolonged warm ischemia time on graft microcirculation. Prior clinical investigations have shown that extended warm ischemia time is closely associated with increased inflammatory responses and subsequent graft dysfunction, a finding that our study reinforces (36). The data suggest that prolonged warm ischemia may exacerbate endothelial cell damage during reperfusion, thereby enhancing the susceptibility of the graft to injury mediated by AT1R antibodies.

Our investigation also delved into the role of complement activation as indicated by C4d deposition in peritubular capillaries. Although C4d positivity has been traditionally regarded as a highly specific marker for antibody-mediated rejection, our results reveal that a notable subset of biopsies with clear microvascular inflammation did not exhibit corresponding C4d staining (37, 38). However, in the group with microvascular inflammation, C4d staining was stronger and more often observed than in the group without it, indicating a potential association. This discrepancy suggests that while C4d is specific, it may lack sufficient sensitivity to capture all instances of antibody-mediated injury—particularly those driven by non-HLA antibodies such as AT1R abs. These findings imply that complement-independent mechanisms may be involved, underscoring the importance of a broader diagnostic approach that incorporates detailed profiling of non-HLA antibodies alongside traditional histopathologic evaluation.

To the best of our knowledge, this study is the first to specifically investigate the incidence of AT1R abs in patients with microvascular inflammation (MVI) using artificial intelligence-based analytical tools. Our approach incorporated correlation analysis, visual inspection using regression plot, logistic regression modelling and association rule mining. While existing literature does not include research focused solely on AT1R abs in this context, AI has been extensively used to predict kidney graft outcomes and episodes of rejection (11).

Numerous machine learning techniques—such as Random Survival Forests, Elastic Net, and deep neural networks—have already proven effective in identifying key predictors of graft survival, optimizing immunosuppressive therapy, and supporting donor–recipient allocation (39–43). These models often outperform traditional clinical scoring systems by uncovering complex, non-linear relationships between variables that may otherwise go undetected. In recent article, AI has also been applied to develop virtual biopsies, enhance histopathological evaluations, and individualize post-transplant immunosuppression strategies, underscoring its broad and evolving role in transplant medicine (44).

Despite these advancements, the role of AT1R abs has been largely overlooked in AI-based studies, particularly in the context of MVI. Our findings, therefore, not only address a critical gap in current research but also highlight the potential of integrating immunological biomarkers into predictive modeling frameworks. The application of AI in this area can enable a more nuanced understanding of kidney transplant immunopathology.

## 5 Conclusions

Our study provides evidence that AT1R antibodies may contribute to microvascular inflammation and subsequent graft injury in kidney transplantation. AI analysis with utilization of association rule mining demonstrated that values of AT1R abs exceeding 12 U/ml were particularly associated with MVI, while lower titers did not show the meaningful association. Our study also explored the role of complement activation through C4d staining and hypothesised that C4d alone may miss some antibody-mediated injuries—especially those caused by non-HLA antibodies like those directed against AT1R. These findings highlight the need for expanded immunologic monitoring beyond traditional HLA antibody screening. Future studies should focus on validating integrated diagnostic models that combine these parameters, which could ultimately lead to more targeted therapeutic interventions and improved long-term allograft survival.

Moreover, by AI tools we subsequently underlined a crucial role of AT1R antibodies in MVI. Looking forward, incorporating AT1R antibodies into larger, multicenter AI-driven datasets could help validate their clinical relevance and prognostic value. Such efforts may ultimately contribute to more precise risk stratification, guide individualized treatment strategies, and improve long-term transplant outcomes.

## 6 Limitations

The study has several important limitations that warrant consideration. Most notably, its retrospective design and lack of pre-transplant non-HLA antibody titers restricts the ability to establish causality between AT1R antibody presence and MVI, particularly given the complex, potentially bidirectional relationship between the two. While the findings support an association, the temporal sequence, whether AT1R antibodies lead to MVI or result from it, remains unresolved. A more informative approach would have involved prospective stratification of patients by AT1R antibody status prior to rejection events, allowing clearer insight into their predictive value. Furthermore, while statistical associations were observed, they appear largely driven by a small subset of patients with very high AT1R abs levels, raising concerns about the generalizability of the findings. The weak correlation coefficients and lack of clear trends among the majority of patients suggest limited explanatory power. Additionally, the commonly used AT1R abs positivity threshold (>10 U/mL) lacks validation for transplant rejection contexts, and over half of MVI patients were antibody-negative, calling into question the biomarker’s sensitivity. Although the study highlights the limitations of C4d staining in detecting non-HLA-mediated injury, it focuses primarily on one aspect of a multifaceted pathophysiological process and does not fully address the dynamic interplay between antibody formation and endothelial damage. Finally, no data on donor-specific antibodies (DSA) was included in the analysis, which is an important limitation, given that this is a strong risk-factor for the occurrence of immunological rejection. Hence, we recommend that the AT1R abs can potentially be an complementary marker for MVI, together with other already existing methods. Nonetheless, more studies on that subject are required to understand the role of AT1R abs in MVI fully.

## Data Availability

The data analyzed in this study is subject to the following licenses/restrictions: The data is available in archives of the University Clinical Hospital in Wroclaw. Due to the privacy policy and General Data Protection Regulations in Poland and European Union the data was not published. Requests to access these datasets should be directed to jakub.mizera@student.umw.edu.pl.
